# A Case of Hepatic Abscess

**Published:** 1853-10

**Authors:** H. R. Casey

**Affiliations:** Columbia county, Ga.


*A Case of Hepatic Abscess.* By H. R. Casey, M.D., of Columbia county, Ga.—I was called to see Mrs. J., æt. 24, whom, upon examination, I found in bed with a face very much flushed, and in a profuse perspiration, high fever, pulse 120, full and bounding. On inquiry, I learned that she had been nursing a case of typhoid fever. She seemed much frightened, thinking that she had contracted that much-to-be-dreaded fever. I soon, however, quieted her fear on that score, telling her that she had no symptom of typhoid, but a fever of an exactly opposite type. I bled her freely, gave a mercurial cathartic, and left fifteen grains quinine, to be administered should the fever subside.

Visited her the next day, when the true character of her disease began to show itself. Pulse 125, tongue heavily coated, skin hot and dry, and slightly tinged with yellow. She now complained of pain in her right side. On examination, I found considerable swelling, the intercostal spaces indicating pressure from within. Ordered leeches over the region of the liver, calomel and Dover's powders in small and separate doses, to be followed with Seidlitz powders; warm cataplasms to succeed the leeches.

Did not see her the next day, from press of business. The following day was summoned to see her in haste. Found her complaining of increased pain and soreness — the distension much greater. There was now evidently a change in the character of her fever; the bright hectic now mantled her cheek, while the blood which had before coursed rapidly along the vessels, now took on a slow and measured step. It was now apparent that the hepatic inflammation had passed into the suppurative stage, and that an abscess was there formed. The liver was very much enlarged, but as yet no pointing. I applied a large blister over the swelling, put her upon a generous diet, and gave her the muriated tinct. of iron, and withheld the lancet for further developments.

Some twenty-four hours thereafter I was again called in haste to see her, when she told me that the pain had left her side, and had located itself in her hip. On examination, I found a spot larger than the palm of my hand on the right hip, fiery red. Not knowing how to account for this, but supposing it to be of an erysipelatous character, I scarrified it, and ordered it to be covered with althea poultices. Continue the tr. ferri.

In the course of the next day I again saw her: found her hip much swollen and fluctuation evident. Continued the application, and on the following morning I plunged my lancet in the tumor, and gave exit at once to at least a pint of matter. It was pus, of a creamy appearance and consistence, with not even a tinge of yellow, and exceedingly offensive. The amount of matter, and the recent date of the hip affection, determined its origin in the liver; but why the pointing should be down here, instead of opposite the liver, I could not answer. This being my first case of hepatic abscess, I was not well "*posted up*"—I judge they are not of frequent occurrence in this country, not remembering an instance where an autopsy has revealed a cicatrix of the liver as the result of hepatic abscess. As further confirmatory of the fact that the matter came from the liver, when the patient sat up, the matter would gravitate in a bolder stream, and by pressing over the liver, and passing the pressure downwards towards the orifice, the matter would make its exit freely. In the progress of this, another pointing was observed opposite the liver, and with my lancet I gave exit to about a teacupful of matter.

Louis states that he has never known of the occurrence of a cicatrix of the liver the result of a cured hepatic abscess. I am certain, if ever the body of this patient becomes a subject of the dissecting knife, a *large* cicatrix of the liver will be among the autopsical appearances.

I called to-day to see the lady, to question her as to the facts of the case. She states that the matter was discharging three months or more, and that she thinks as much as three gallons of it passed off in the time. This, of course, is too large an estimate. She states that she is in the enjoyment of good health, but has occasionally pains in her right side, when she stoops down or bends to the opposite side, doubtless from adhesions of the liver. She thinks her "entrails have grown to her side," and states that she was "physicked" some years since for liver disease.—*Southern Med. and Sur. Jour.*

